# Overview of the Clinical Potential of microRNAs in the Detection of Suicide

**DOI:** 10.7759/cureus.94501

**Published:** 2025-10-13

**Authors:** Karla-Maria Lopez-Martinez, Jose Luis Cortez-Sanchez, Gilberto Perez Sánchez, José Miguel Chin Chan, Elizabeth Bautista-Rodriguez

**Affiliations:** 1 Faculty of Biotechnology, Universidad Popular Autónoma del Estado de Puebla, Puebla, MEX; 2 Faculty of Chemical and Biological Sciences, Universidad Autónoma de Campeche, Campeche, MEX; 3 Psychoimmunology Laboratory, Ramón de la Fuente Muñíz National Institute of Psychiatry, Mexico City, MEX; 4 Environmental Epigenetics and Mental Health Laboratory, Faculty of Chemical Biological Sciences, Universidad Autónoma de Campeche, Campeche, MEX; 5 Faculty of Health Sciences, Universidad Autónoma de Tlaxcala, Tlaxcala, MEX

**Keywords:** biomarkers, major depressive disorder (mdd), micrornas (mirnas), suicidal behavior, epigenetics

## Abstract

Suicide represents a priority clinical challenge because of the lack of specific biological and molecular biomarkers. The development of such markers would, together with clinical findings, allow for a concrete diagnosis. MicroRNAs (miRNAs), because of their stability in biological fluids and their role in gene regulation, emerge as useful candidates to complement traditional clinical evaluation. This narrative review synthesizes evidence on the role of miRNAs as biomarkers in suicide and suicidal behavior, based on reports in brain tissue and fluids such as peripheral blood, integrating data on miRNA regulation and their associated molecular pathways. Several miRNAs, including miR-124, miR-18a, miR-132, miR-185, miR-218, and miR-19a-3p, are consistently dysregulated across multiple studies. These findings suggest that miRNA measurement could complement clinical scales and provide an objective biological marker for suicide risk. Their integration into diagnostic protocols could improve risk stratification and open the door to personalized prevention strategies; however, it is essential to validate miRNA panels in multicenter clinical studies before routine implementation.

## Introduction and background

Suicide is defined as any self-induced action that results in the death of the individual, this being the intended outcome of the act [1]. The World Health Organization (WHO) identifies suicide as a major public health problem, being the fourth leading cause of death among young people aged 15 to 19, and reports that approximately 800,000 people die by suicide every year, representing one in every 100 deaths worldwide [2]. Suicidal behavior, which includes ideation, planning, and attempts, is a complex and multifactorial phenomenon associated with psychiatric, psychological, biological, and environmental factors [3,4].

Understanding suicide risk at the biological level is a growing area of research, with biomarkers emerging as promising tools for assessment and intervention; the search for suicide biomarkers has emphasized neurotransmitters (such as serotonin, dopamine, and gamma-aminobutyric acid (GABA)), the hypothalamic-pituitary-adrenal (HPA) axis, neurotrophic factors such as brain-derived neurotrophic factor (BDNF), and intracellular signaling pathways as mitogen-activated protein kinase (MAPK), Wingless and Int-1 (Wnt), and cAMP-responsive element binding protein 1 (CREB1) [5,6]. In addition, microRNAs (miRNAs), short, non-coding RNAs that regulate gene expression at the post-transcriptional level, are emerging as valuable biomarkers and potential therapeutic targets in both psychiatric and other medical conditions [7,8].

This narrative review aims to provide an overview of the role of miRNA dysregulation in suicide and suicidal behavior by addressing the miRNAs that are most consistently dysregulated, their proposed roles in the underlying neurobiology, and their potential for developing into clinically applicable biomarkers.

## Review

MicroRNAs (miRNAs)

miRNAs are small non-coding single-stranded type of RNAs, of 18-22 nucleotides in length, endogenous and highly conserved across species. miRNAs are transcribed in the nucleus, processed by enzymes (Drosha and Dicer), to then be exported as mature miRNAs to the cytoplasm, to be incorporated in the RNA-induced silencing complex (RISC), which is guided to the target mRNA, suppressing gene expression [9].

The primary function of miRNAs is gene regulation through mRNA degradation and by controlling transcription and translation [10]. According to Chevillet et al.'s [11] literature review, a single miRNA could potentially target numerous mRNAs, while at the same time, an mRNA could contain multiple binding sites for miRNAs, allowing many biological processes to be regulated by this interaction. Among other physiological roles, miRNAs are involved in cell division, differentiation, apoptosis, and immune processes; and its dysregulation has been linked to several diseases [12].

However, the most promising role of miRNAs is as biomarkers, due to their stability in body fluids, facilitating non-invasive diagnosis and prognosis [9]. Biomarkers can provide information useful for diagnosis, prognosis, and treatment of various diseases [10]. miRNAs are no exception; their advantage as biomarkers lies in their potential use for precise diagnosis, guided treatment, and evaluation of treatment response [9].

Knowing the specific targets of each miRNA, identifying their objectives, and understanding the mechanisms regulating them has helped clarify their biological roles and their involvement in the development of various pathologies [13]. Different studies have concluded that miRNAs regulate the expression of their targets; consequently, deregulation of miRNA functions can lead to disease development.

The presence of miRNAs in serum is well documented, along with at least 12 other fluids such as plasma, saliva, tears, and urine [14]. It has also been shown that, depending on the fluid, between 200 and approximately 450 miRNAs may be detected. These circulating miRNAs are more resistant and less labile, making them less likely to degrade in the extracellular environment [8]. Different methodologies have been described for identifying miRNAs in various diseases, with quantitative reverse transcription polymerase chain reaction (RT-qPCR) using TaqMan probes being the most widely used for its sensitivity and specificity; other methods include northern blot hybridization (which requires larger amounts of total RNA), in situ hybridization (to compare expression levels across different cell types), next-generation sequencing (highly sensitive but expensive), genomic profiling, and more recently microarrays [8,10].

miRNAs in suicide and suicidal behavior

miRNAs in the central nervous system (CNS) account for approximately 70% of all miRNAs in the human body. They play roles in regulating neurogenesis and neuroplasticity, both of which undergo significant changes in suicidal behavior and psychiatric disorders, including suicide [8].

In human brains, particularly in the dorsolateral and ventral prefrontal cortex, downregulation of the miR-183/96/182 cluster and miR-146a/b has been observed, affecting MAPK, Wnt, and CREB1 signaling pathways, all of which are associated with synaptic plasticity [15,16]. Likewise, overexpression of miR-185 and decreased levels of miR-218 are associated with dysfunction in BDNF/tyrosine receptor kinase B (TrkB) and netrin-1 receptors (DCC) signaling, respectively, supporting the hypothesis of impaired survival and reduced neuroprotection [17,18].

Other relevant findings come from the locus coeruleus, where altered miRNA networks regulating genes such as RELN, GSK3β, and MAOA were identified, linking gene dysregulation with major depression and anxiety, both risk factors for suicidal behavior [19]. Furthermore, miRNAs detected in peripheral blood, such as miR-19a-3p, a regulator of TNF-α, suggest a convergence between inflammatory processes and suicide risk, as with the added value of their potential as clinically accessible circulating biomarkers [20].

Table 1 lists the main miRNAs described to date as directly related to suicide and suicidal behavior, suicide victims, and suicide victims with major depressive disorder (MDD). In specific situations, their regulation is altered - either upregulated (higher levels in the analyzed tissue) or downregulated (lower levels) - when compared to levels found in clinically healthy individuals. Their target genes or pathways are also described, which have previously been associated with suicide or suicidal behavior.

**Table 1:** miRNAs in suicide and suicide ideation Selected high-impact studies showing miRNA alterations in suicidal victims and suicidal ideation patients with or without major depressive disorder (MDD). References: [15,16,17,18,19,21,22,23,24]

Author	miRNAs	Regulation	Study subjects	Target (gene/pathway)	Tissue	Association/clinical implication
Smallheiser et al., 2012	miR-142-5p, miR-137, miR-489, miR-148b, miR-101, miR-324-5p, miR-301a, miR-146a, miR-335, miR-494, miR-20b, miR-376a, miR-190, miR-155, miR-660, miR-130a, miR-27a, miR-497, miR-10a, miR-20a, miR-142-3p	Downregulation	MDD suicide victims (n = 35)	DMNT3b, VEGFA BCL2	Prefrontal cortex (BA9)	Association between miRNAs and depression
Maussion et al., 2012	miR-185	Upregulation	Suicide victims (n = 38)	TrkB-T1	Prefrontal cortex	Altered BDNF-mediated neuroplasticity
Roy et al., 2017	miR-17-5p, -20b-5p, -106a-5p, -330-3p, -409-5p, -541-3p, -582-5p, -890, let-7g-3p, -99, -550-5p, -1179	Downregulation	Suicide victims with MDD (n = 9)	RELN, GSK-3β, MAOA, CHRM1, PLCB1, GRIK1	Locus coeruleus	miRNA networks associated with major depression and anxiety
Roy et al., 2017	miR-409-5p, let-7g-3p, miR-1197	Upregulation	Suicide victims with MDD (n = 9)	RELN, GSK-3β, MAOA, CHRM1, PLCB1, GRIK1	Locus coeruleus	Altered regulation of neuronal networks
López et al., 2017	miR-146a-5p, -146b-5p, -425-3p, -24-3p	Downregulation	MDD patients (n = 258)	MAPK, Wnt pathways, cell adhesion	Ventral prefrontal cortex	Association with major depression and neuroinflammation
Torres-Berrío et al., 2017	miR-218	Downregulation	Suicide victims with MDD (n = 35)	DCC	Prefrontal cortex	Vulnerability to depression and suicidal behavior
Wang et al., 2018	miR-19a-3p	Upregulation	Suicide victims and suicide ideation (n = 55)	TNF-α	Frontal cortex and peripheral blood	Associated with inflammation and suicide risk
Gorinski et al., 2019	miR-30e	Upregulation	Suicide victims with MDD (n = 32)	ZDHHC21	Dorsolateral prefrontal cortex	Altered BDNF-mediated neuroplasticity
Stapel et al., 2022	miR-30a, miR-30e, and miR-200a	Upregulation	MDD with suicide ideation	EGR1, MBNL1, RARB and REEP3	Serum	Differences between recent attempts and the history of attempts
Kosten et al., 2022	miR-424-5p, miR-378i, miR-6724-5p, and miR-10b-5p	Downregulation	Suicide ideation (n = 42)	MAPK, ErbB, AMPK, Ras, p53, and PI3K-Akt	Plasma	miRNAs differential expression after SI recovery after four to six weeks

miRNAs in suicide and stress

Several studies have directly linked miRNAs to these disorders. Suicidal behavior has been increasingly associated with dysregulation of the hypothalamic-pituitary-adrenal (HPA) axis, the body's central stress response system [25]. Studies suggest that both an overactive and an underactive HPA axis can elevate suicide risk, with genetic predispositions, epigenetic modifications, and environmental influences all contributing to this complex relationship [25,26]. This type of stress alters several miRNAs during brain maturation; therefore, it is theorized that early trauma produces changes in miRNAs, which can be modulated by antidepressants [7].

The influence of stress on miRNA expression across various brain regions is a key area of investigation. Certain miRNAs, notably miR-185 and miR-491-3p, have been identified as important players in major depression and suicide. miR-185's function is particularly relevant as it targets TrkB isoform T1, a receptor that facilitates neurotrophic signaling and stress response, thereby creating a potential link to HPA axis function [17,26].

miRNAs in suicide and psychiatric disorders

It is well-documented that miRNAs play a significant role in biogenesis and brain function; it has been described that specific miRNAs (e.g., miR-9, miR-124, miR-7, and miR-135) act as master regulators or fine-tuners, targeting multiple mRNAs to coordinate complex developmental programs and maintain neuronal identity, regulating synaptic function, memory formation, and cognitive processes by controlling local protein synthesis and neuronal excitability [12]; also, they have been implicated in the development of psychiatric disorders, especially those associated with suicidal behavior like schizophrenia, bipolar disorder, and MDD [27]. In schizophrenia, there is a strong association with miR-137 [28,29]. Other miRNAs are also involved, such as upregulation of miR-34a, miR-132, and miR-212 and downregulation of let-7 family members and miR-181b [30-32]. In addition, several miRNAs have been associated with suicidal behavior in schizophrenia, impacting synaptic plasticity and communication, including upregulated miR-376a and miR-625 and downregulated miR-152, miR-181a, miR-330-3p, miR-34a, and miR-133b [17,33].

In bipolar disorder (BD), which shares genetic overlap with schizophrenia, some miRNAs are similarly dysregulated. For example, miR-154 is downregulated in both disorders [34]. In addition, miR-34a is upregulated; elevated levels disrupt neural morphology and differentiation, and this miRNA has also been reported in BD patients who died by suicide [35]. The upregulation of miR-185 (which targets TrkB-T1) and the downregulation of synaptic miRNAs, such as miR-152, as observed in synaptosome studies, are also highly relevant for BD patients who died by suicide [17,33].

MDD is strongly associated with suicide and represents the psychiatric condition with the highest risk of suicide. Several mechanisms are affected, including inflammation, where miR-19a-3p is upregulated. This miRNA targets the pro-inflammatory cytokine TNF-α, and its upregulation leads to increased TNF-α expression and neuroinflammation, a key proposed mechanism in suicidal behavior [19,21]. In synaptic function, miR-185 and miR-491-3p are upregulated; miR-185 reduces the expression of TrkB-T1, a truncated isoform of the BDNF receptor, an alteration commonly found in both MDD and suicide [17]. Upregulation of miR-34c-5p, miR-320c, and miR-139-5p has been correlated with downregulation of the polyamine metabolism genes SAT1 and SMOX, which are involved in cellular stress response; this directly links miRNA dysregulation to stress-related pathology in suicide [36]. Some studies have also found downregulation of miR-34b-5p and miR-369-3p in leukocytes of MDD patients with suicidal ideation compared to those without [37].

miRNAs in suicide and childhood trauma

Childhood trauma is a well-established and potent risk factor for suicidal ideation, attempts, and behaviors across the lifespan. Research consistently shows that various forms of early adversity, including abuse and neglect, along with genetic factors, significantly increase vulnerability to suicide, with effects persisting into adolescence, adulthood, and late life by altering neural plasticity and stress response systems [38,39].

Several miRNAs (e.g., miR-497, miR-146b-5p, miR-330-3p) have been found to be altered in both individuals with early life stress (ELS) and in the brains of suicide victims, indicating possible shared molecular pathways [39]. In patients with MDD, miR-17 and miR-92 are specifically associated with a history of childhood trauma, suggesting these miRNAs may be involved in the pathophysiology linking trauma to depression and suicide risk [40].

miRNAs dysregulated by childhood trauma often regulate genes involved in neural signaling, stress response, and synaptic plasticity, which are critical for emotional regulation and resilience [39,40]. Some miRNAs (e.g., miR-641) are linked to impulsivity, a trait associated with both ELS and increased suicide risk [39]. Changes in miRNA expression may interact with other biological systems, such as the HPA axis and immune response, further influencing suicide vulnerability [41,42].

miRNAs as clinical biomarkers in suicide

Post-mortem studies of suicide victims, analyzing the entire miRNA expression profile, have shown dysregulation in the overall expression of miRNAs, which may serve as a molecular pattern or signature [26]. Twenty-one significantly dysregulated miRNAs were identified, most of which had a nearby chromosomal localization, suggesting coordinated regulation and that they were possibly transcribed by the same pri-miRNA transcripts, indicating that the dysregulated expression of miRNAs was due to decreased transcription [15]. The regulatory potential of miRNAs is vast; the fact that one miRNA can target many genes and one gene can be regulated by many miRNAs enables the creation of a complex regulatory network, this network frequently bears a disease-specific molecular signature shaped by pathological alterations, as observed in suicidal behavior [11].

As summarized in Table 1, various studies have pinpointed specific miRNAs in blood components such as plasma and serum. A key advantage of these molecules is their capacity to cross the blood-brain barrier, allowing peripheral expression levels to provide a window into central nervous system processes; consequently, circulating miRNAs represent a promising class of non-invasive biomarkers for identifying individuals at risk for suicidal behavior. This has been shown by Wang et al. [21], when they described miR-19-3p upregulation in prefrontal cortex (suicide victims) and in peripheral blood (suicide ideation), which is linked to immune and serotonin-related pathways. In Stapel et al.'s clinical study [23], miR-30a, miR-30e, and miR-200a were significantly elevated in the serum of patients with MDD who were at acute suicide risk or had recently attempted suicide. Their levels are higher in those with recent suicide attempts compared to those with only suicidal ideation, suggesting their potential as trait markers for suicidal behavior [23]. Kosten et al. found that miR-424-5p, miR-378i, miR-6724-5p, and miR-10b-5p decreased plasma levels in patients recovering from strong suicidal ideation, indicating their potential role in monitoring recovery [24], along with Sun et al.'s study, showing the downregulation of miR-34b-5p and miR-369-3p in leukocytes of MDD patients with suicidal ideation [37].

The consistent dysregulation of specific miRNAs like miR-185 and miR-19a-3p in key pathways involved in neuroplasticity, inflammation, and stress response implies a central role for them in the molecular pathology of suicide. This, coupled with their stability in peripheral blood, positions them as promising non-invasive biomarkers capable of providing an objective window into brain-related pathology and potentially identifying individuals at acute risk, thereby addressing a major clinical challenge. Future research must prioritize large-scale validation and standardization of detection methods to translate these findings into clinical tools.

## Conclusions

Collectively, the evidence firmly establishes dysregulated miRNAs as a promising molecular signature of suicide, suicide risk, and ideation. Their ability to regulate complex gene networks involved in synaptic plasticity, MDD and anxiety, stress response systems, and immune-inflammation mechanisms positions them as key players in the underlying pathology. Crucially, their stability and detectability in blood make them viable non-invasive biomarkers, offering a potential tool for objective risk assessment that could revolutionize early identification and preventive strategies.

For this potential to be validated, future research must address current limitations through large-scale, longitudinal studies. The primary goal is to validate standardized miRNA panels across diverse populations, which is an essential step toward their eventual integration into clinical practice for suicide prevention.
